# The Effects of Vitamin D Receptor Silencing on the Expression of LVSCC-A1C and LVSCC-A1D and the Release of NGF in Cortical Neurons

**DOI:** 10.1371/journal.pone.0017553

**Published:** 2011-03-03

**Authors:** Duygu Gezen-Ak, Erdinç Dursun, Selma Yilmazer

**Affiliations:** Istanbul University, Cerrahpasa Faculty of Medicine, Department of Medical Biology, Istanbul, Turkey; The Scripps Research Institute, United States of America

## Abstract

**Background:**

Recent studies have suggested that vitamin D can act on cells in the nervous system. Associations between polymorphisms in the vitamin D receptor (VDR), age- dependent cognitive decline, and insufficient serum 25 hydroxyvitamin D_3_ levels in Alzheimer's patients and elderly people with cognitive decline have been reported. We have previously shown that amyloid β (Aβ) treatment eliminates VDR protein in cortical neurons. These results suggest a potential role for vitamin D and vitamin D-mediated mechanisms in Alzheimer's disease (AD) and neurodegeneration. Vitamin D has been shown to down-regulate the L-type voltage-sensitive calcium channels, LVSCC-A1C and LVSCC-A1D, and up-regulate nerve growth factor (NGF). However, expression of these proteins when VDR is repressed is unknown. The aim of this study is to investigate LVSCC-A1C, LVSCC-A1D expression levels and NGF release in VDR-silenced primary cortical neurons prepared from Sprague-Dawley rat embryos.

**Methodology/Principal Findings:**

qRT-PCR and western blots were performed to determine VDR, LVSCC-A1C and -A1D expression levels. NGF and cytotoxicity levels were determined by ELISA. Apoptosis was determined by TUNEL. Our findings illustrate that LVSCC-A1C mRNA and protein levels increased rapidly in cortical neurons when VDR is down-regulated, whereas, LVSCC-A1D mRNA and protein levels did not change and NGF release decreased in response to VDR down-regulation. Although vitamin D regulates LVSCC-A1C through VDR, it may not regulate LVSCC-A1D through VDR.

**Conclusions/Significance:**

Our results indicate that suppression of VDR disrupts LVSCC-A1C and NGF production. In addition, when VDR is suppressed, neurons could be vulnerable to aging and neurodegeneration, and when combined with Aβ toxicity, it is possible to explain some of the events that occur during neurodegeneration.

## Introduction

The name “vitamin D” is a misnomer; in actuallity, vitamin D (1,25 (OH)_2_D_3_) is a multipurpose secosteroid hormone. It consists of a broken cholesterol backbone, and it has steroid-like effects, such as regulating the expression of over 1,000 genes. Although a relatively limited number of studies have investigated the genes targeted by vitamin D in the brain [1], [2], [3], [4], [5], [6], [7], [8], [9], [10], [11], [12], [13], [14], the probable effects of vitamin D on neurotrophic factor production, oxidative stress mechanisms, Ca^2+^ homeostasis and the immune system are irrefutable. A study on hippocampal neuron cultures suggest that vitamin D promotes calcium homeostasis by decreasing the level of L-type voltage-sensitive calcium channels (LVSCC), and channel density, on the plasma membrane [2]. In addition, vitamin D is involved in determining neuronal fate via its regulation of nerve growth factor (NGF) expression [8].

Vitamin D exerts its effects through its nuclear hormone receptor, vitamin D receptor (VDR) or its membrane receptor, membrane-associated, rapid-response, steroid-binding protein (1,25 MARRS) [15]. Recently, it was shown that VDR and the enzymes involved in bioactivation of vitamin D, are abundantly expressed in the majority of the central nervous system, particularly in areas affected by neurodegenerative disorders [5], [8], [16], [17], [18].

Consistent with its expression pattern, there is a correlation between polymorphisms inVDR, age-dependent cognitive decline and insufficient vitamin D precursor (25 hydroxyvitamin D_3_) levels in serum from AD patients and elderly people with cognitive decline [19], [20], [21], [22], [23]. We have previously shown that polymorphisms in *VDR* may increase the vulnerability to Alzheimer's disease (AD) [24]. In addition, we have shown that amyloid β (Aβ) treatment eliminates expression of VDR mRNA and protein in cortical neurons [25]. Furthermore, we have shown that vitamin D can protect neurons against Aβ-induced toxicity by down-regulating LVSCC-A1C expression, up-regulating VDR expression and inducing NGF release [25]. These results indicate that vitamin D and vitamin D-related mechanisms may function in AD and neurodegeneration.

Our aim is to determine whether down-regulation of VDR leads to alterations in the expression of calcium channels and neurotrophic factors in neurons. To investigate the effects of VDR down-regulation in some of the neurodegeneration-related mechanisms, VDR was knocked down in cultured rat cortical neurons using small interfering RNA (siRNA) induced gene silencing. The effects on LVSCC-A1C and LVSCC-A1D expression and NGF levels were investigated after siRNA treatment to determine the effect that disruption of the vitamin D-VDR pathway has on these proteins, and whether vitamin D-induced regulation of these proteins depends on VDR.

## Results

Previously, we demonstrated that vitamin D treatment up-regulates VDR, down regulates LVSCC-A1C and induces NGF release in cortical neurons [25]. Prior to this study we showed that expression of LVSCC-A1D mRNA was down-regulated in vitamin D-treated cortical neurons compared to the untreated control neurons (p = 0.005). Our primary goal in the present study is to investigate alterations of the LVSCC-A1C, LVSCC-A1D and NGF in VDR-silenced neurons.

### Cytotoxicity and Cell Death Assay

Cytotoxicity and cell death assays were used to determine the overall effect of short-term VDR silencing on neuronal survival. Lactate dehydrogenase (LDH), which is an enzyme released in the extracellular environment in response to membrane damage and/or oxidative stress-related cell death, was measured to determine cytotoxicity levels. Terminal deoxynucleotidyl transferase dUTP nick end labeling (TUNEL) was used to determine whether cell death occurred as a result of apoptosis.

There was no significant difference in LDH release between siRNA-treated groups and control groups in cortical neurons after 24 hours of treatment. No significant difference was observed in the apoptotic index between siRNA-treated groups and control groups in cortical neurons after 24 hours of treatment. These results indicated that siRNA treatment did not cause cytotoxicity or apoptosis.

### qRT-PCR results

Quantitative real time-polymerase chain reaction (qRT-PCR) was used to determine the effects of all treatments on the relative expression of target genes by measuring mRNA levels. To determine the specificity of VDR silencing, and the rate of silencing, three additional control groups [Cyclophiline B (CycB) siRNA, non-target siRNA and transfection reagent] were used to treat neurons, in addition to untreated control neurons.

**Figure 1:**
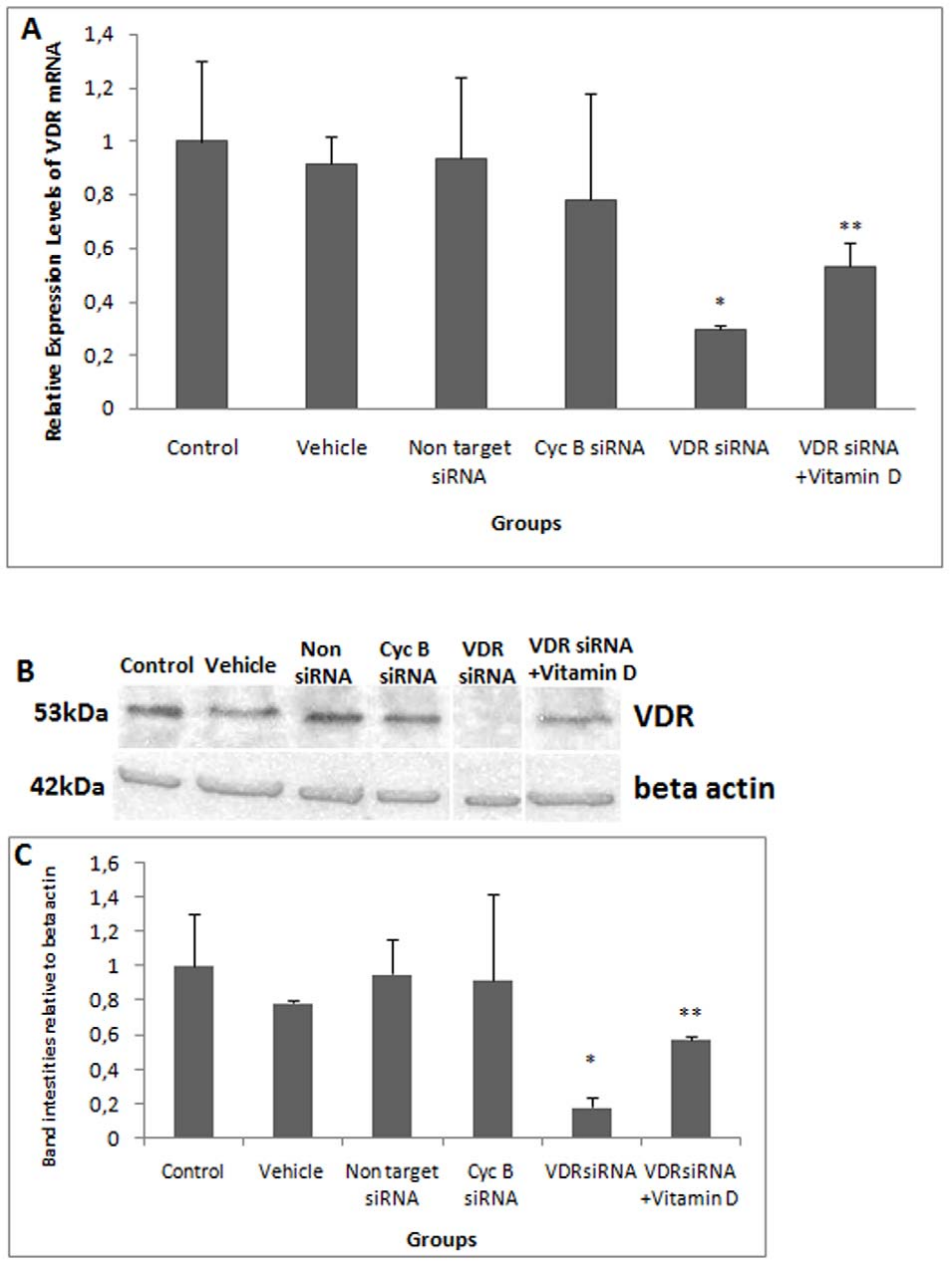
siRNA-mediated knockdown of VDR. **A) Comparison of VDR mRNA levels.** VDR siRNA treatment suppressed VDR mRNA expression. After 12 hours of vitamin D treatment (1×10^−7^ M) applied to VDR-silenced neurons, VDR mRNA levels increased. These results indicate that vitamin D increases VDR expression in cortical neurons * VDR mRNA levels from VDR-silenced neurons were statistically lower than in the control groups (p<0.001, p<0.001, p<0.001, p<0.001, respectively). ** VDR mRNA levels from Vitamin D-treated VDR-silenced neurons were statistically higher than in the VDR siRNA-treated group (p<0.001). **B) Western blot detecting VDR protein level.** VDR siRNA treatment suppressed VDR protein expression. After 12 hours of vitamin D treatment (1×10^−7^ M) in VDR-silenced neurons, VDR protein levels increased. Beta actin was used as the loading control. **C) Comparison of VDR protein band intensities relative to Beta actin.** The absolute intensities of VDR and Beta actin protein bands were measured using Image J software, and the relative intensities were calculated from the ratio of VDR to Beta actin absolute intensities. * VDR protein levels from VDR-silenced neurons were statistically lower than in control groups (p<0.001, p<0.001, p<0.001, p<0.001, respectively). ** VDR protein levels from vitamin D-treated VDR-silenced neurons were statistically higher than in the VDR siRNA-treated group (p<0.05). *Control*: Untreated control group; *Vehicle*: Transfection reagent-treated control group; *Non target siRNA:* Non-target siRNA-treated negative control group; *Cyc B siRNA*: Cyclophilin B siRNA-treated positive control group; *VDR siRNA*: VDR siRNA-treated group and *VDR siRNA+Vitamin D*: Following 12 hours of VDR siRNA treatment, groups were treated with vitamin D. Data are presented as a mean SD.

**Figure 2:**
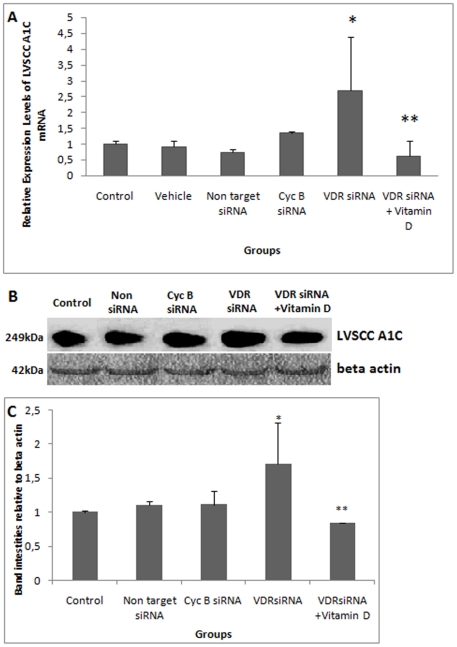
siRNA-mediated knockdown of VDR induces expression of LVSCC-A1C mRNA and protein. A) Comparison of LVSCC-A1C mRNA levels. VDR suppression resulted in increased LVSCC-A1C mRNA expression, but the effects of VDR suppression on LVSCC-A1C were normalized after vitamin D treatment.* LVSCC-A1C mRNA levels from VDR-silenced neurons were statistically higher than in other groups (p = 0,015, p = 0,034, p = 0,002, p = 0,024, respectively). ** LVSCC-A1C mRNA levels were statistically lower than in VDR siRNA-treated group (p = 0,013). **B) Detection of LVSCC-A1C protein by western blot.** Although LVSCC-A1C protein increased in VDR-silenced neurons, vitamin D treatment decreased LVSCC-A1C expression to control levels. Beta actin was used as loading control. **C) Comparison of LVSCC-A1C protein band intensities relative to Beta actin.** Western blot results were consistent with mRNA results. The absolute intensities were measured using Image J software, and the relative intensities were calculated from the ratio of LVSCC-A1C to Beta actin absolute intensities. * LVSCC-A1C protein levels from VDR-silenced neurons were statistically higher compared to control groups (p<0.01, p<0.01, p<0.05, respectively). ** LVSCC-A1C protein levels from vitamin D-treated VDR-silenced neurons were statistically lower compared to the VDR siRNA-treated group (p<0.001). *Control*: Untreated control group; *Vehicle*: Transfection reagent-treated control group; *Non target siRNA:* Non-target siRNA-treated negative control group; *Cyc B siRNA*: Cyclophilin B siRNA-treated positive control group; *VDR siRNA*: VDR siRNA-treated group and *VDR siRNA+Vitamin D*: Following 12 hours of VDR siRNA treatment, groups were treated with vitamin D. Data are presented as a mean SD.

**Figure 3:**
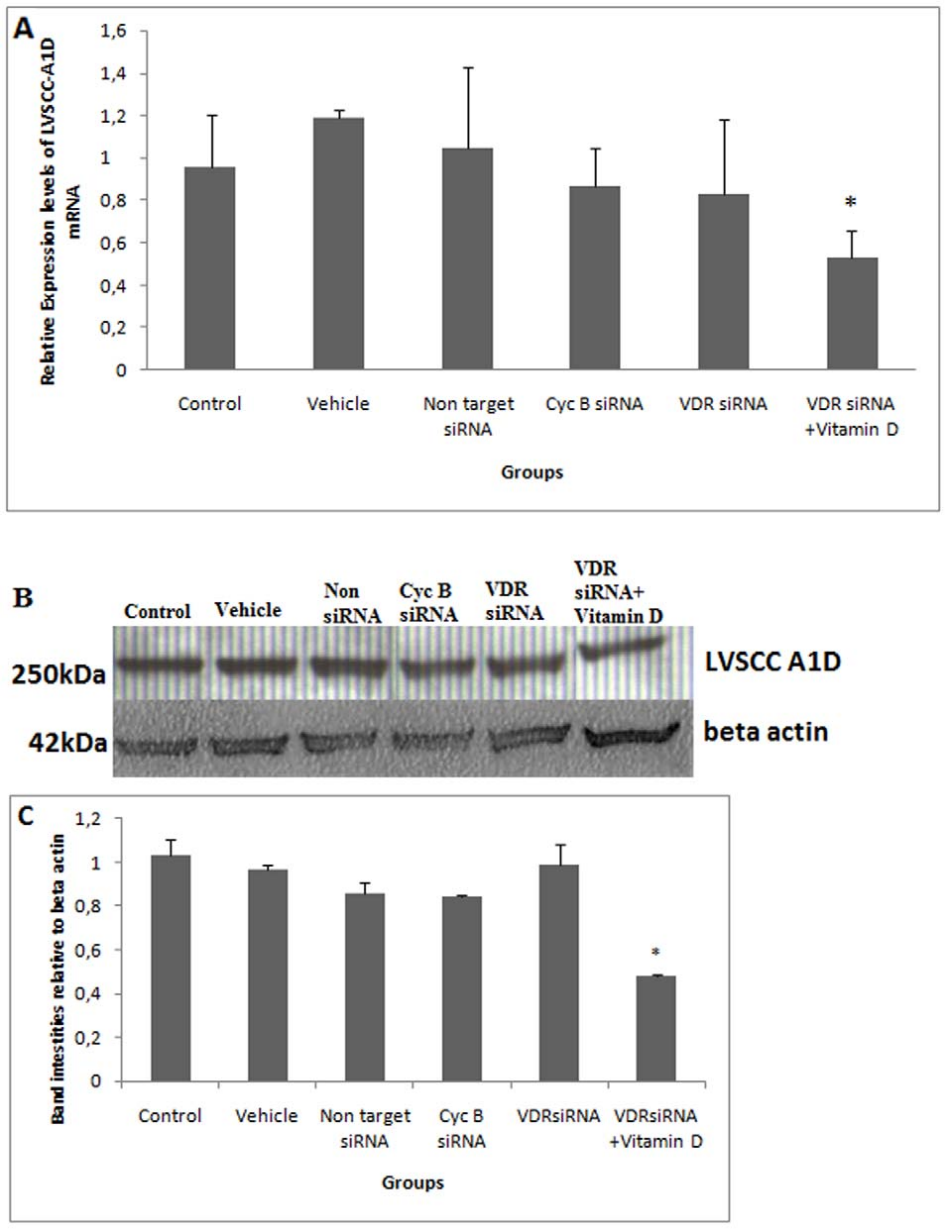
VDR siRNA-mediated knockdown does not change the expression of LVSCC-A1D mRNA and protein. **A) Comparison of LVSCC-A1D mRNA levels.** LVSCC-A1D mRNA levels from VDR-silenced neurons were not different than control groups (p>0.05), but LVSCC-A1D mRNA levels significantly decreased after vitamin D treatment in VDR-silenced neurons. * LVSCC A1D mRNA levels from vitamin D-treated VDR-silenced neurons were statistically lower than control levels (p = 0,019, p = 0,019, p = 0,030, p = 0,013, respectively) and VDR siRNA-treated groups (p = 0,031). **B) Detection of LVSCC-A1D protein by western blot.** LVSCC-A1D protein levels did not change in VDR-silenced neurons. Vitamin D treatment in VDR-silenced neurons led to a decrease in LVSCC-A1D protein levels. Beta actin was used as a loading control. **C) Comparison of LVSCC-A1D protein band intensities relative to beta actin.** Western blot results were consistent with mRNA results. The absolute intensities were measured using Image J software, and the relative intensities were calculated from the ratio of LVSCC-A1D to beta actin absolute intensities. * LVSCC-A1D protein levels from vitamin D-treated VDR-silenced neurons were statistically lower compared to other groups (p<0.001, p<0.001, p<0.001, p<0.001, p<0.001, respectively). *Control*: Untreated control group; *Vehicle*: Only transfection reagent-treated control group; *Non target siRNA:* Non-target siRNA-treated negative control group; *Cyc B siRNA*: Cyclophilin B siRNA-treated positive control group; *VDR siRNA*: VDR siRNA-treated group and *VDR siRNA+Vitamin D*: Following 12 hours of VDR siRNA treatment, groups were treated with vitamin D. Data are presented as a mean SD.

After 6 hours of VDR siRNA treatment, VDR was not silenced. Alterations in LVSCC-A1C mRNA expression were not detected in these groups. These data were not presented. After12 hours of VDR siRNA treatment, VDR mRNA levels were significantly reduced (Fig. 1A). Significantly higher levels of LVSCC-A1C mRNA were observed 12 hours after VDR siRNA treatment (Fig. 2A). After 24 hours of VDR siRNA treatment, there was a significant reduction in VDR mRNA levels and an increase in LVSCC-A1C mRNA levels (Table 1). VDR silencing occurred after both 12 and 24 hours of treatment, and LVSCC-A1C mRNA expression was up-regulated during the same time periods. In contrast, LVSCC-A1D mRNA levels did not change after 12 (Fig. 3A) and 24 hours of treatment (p>0.05). These results indicate that VDR siRNA treatment suppresses VDR expression. Furthermore, VDR suppression results in increased LVSCC-A1C mRNA expression, but does not affect LVSCC-A1D mRNA levels.

**Table 1:** The effects of siRNA-mediated knockdown of VDR onVDR and LVSCC-A1C mRNA expression in 24 hour treatment.

Compared groups	***mRNA Expression	Std. Error	95% C.I.	P(H1)	Result
**VDR mRNA levels of 24 hour treatment**					
Cont vs. VDR siRNA	0.350	0.280–0.451	0.246–0.485	0.000	DOWN*
Vehicle vs. VDR siRNA	0.312	0.208–0.452	0.168–0.603	0.000	DOWN
Non Target vs. VDR siRNA	0.338	0.240–0.459	0.222–0.495	0.000	DOWN
CycB siRNA vs. VDR siRNA	0.386	0.270–0.542	0.255–0.556	0.000	DOWN
**LVSCC-A1C mRNA levels of 24 hour siRNA treatment**					
Cont vs. VDR siRNA	2.992	2.150–4.588	1.677–6.186	0.002	UP**
Vehicle vs. VDR siRNA	2.839	1.849–4.221	1.280–7.994	0.001	UP
Non Target vs. VDR siRNA	3.198	1.631–5.679	1.174–6.847	0.003	UP
CycB siRNA vs. VDR siRNA	1.693	1.064–2.725	0.776–3.825	0.014	UP

REST 2008 was used for statistical analysis.

* VDR is DOWN-regulated in sample group (in comparison to control groups).

** LVSCC-A1C is UP-regulated in sample group (in comparison to control groups).

*** Expression values have normalized to the 3 housekeeping genes, GAPDH, HPRT, ACTB.

Treatment with CycB siRNA (positive control) resulted in significantly lower levels of CycB mRNA. There were no significant differences in VDR, LVSCC-A1C or LVSCC-A1D mRNA levels after 12 and 24 hours of CycB siRNA treatment. These results indicate that alterations in mRNA expression were specific to VDR siRNA treatment.

### Western Blot results

Western blots were used to determine the efficiency of VDR silencing at the protein level and to investigate whether changes in mRNA expression correspond to changes in VDR, LVSCC-A1C and, LVSCC-A1D protein levels.

VDR silencing was confirmed at the protein level (Fig. 1B and 1C). Higher levels of LVSCC-A1C protein were observed after 12 (Fig. 2B and 2C) and 24 hours of VDR siRNA treatment, in agreement with the mRNA results. In addition, LVSCC-A1D protein levels at 12 (Fig.3B and 3C) and 24 hours after VDR siRNA treatment did not change, consistent with the mRNA results. Only results from the 12 hour siRNA treatment were given because the 24 hour siRNA treatment results were similar to the 12 hours results.

### VDR siRNA and vitamin D (1,25 (OH)_2_D_3_) combined treatments

Combined treatments with VDR siRNA and vitamin D were performed to determine the effects of vitamin D treatment on the expression of genes of interest in VDR-silenced neurons.

VDR mRNA and protein levels were up-regulated in the group that received 12 hours of 1×10^−7^ M vitamin D after 12 hours of VDR siRNA treatment (VDR siRNA+ vitamin D) (Fig. 1), whereas LVSCC-A1C (Fig. 2) and LVSCC-A1D mRNA and protein (Fig. 3) levels were down-regulated when compared to the VDR siRNA-treated group. These results indicate that vitamin D leads to an increase in VDR expression in cortical neurons and may rapidly regulate target genes by up-regulating VDR expression after VDR is silenced in neurons.

### NGF Assay

**Figure 4:**
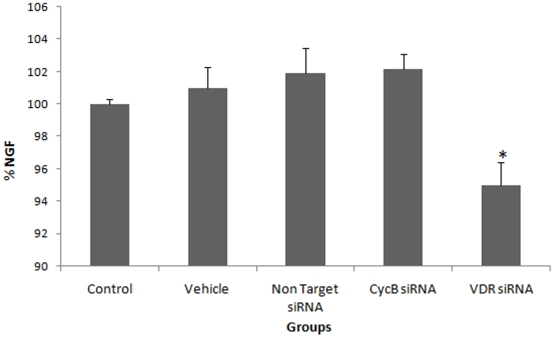
siRNA-mediated knockdown of VDR leads to a significant reduction in NGF release. * VDR-silenced neurons had significantly lower NGF release compared to control, vehicle, non-target siRNA and cycB siRNA-treated groups (p<0.05, p<0.01, p<0.01, p<0.01, respectively). Data are presented as a mean SD.

We also investigated whether VDR regulation correlates with the levels of NGF release when the vitamin D-VDR pathway is blocked. There was no significant difference in NGF protein levels after 12 hours of siRNA treatment (p>0.05). After 24 hours of VDR siRNA treatment, there was a significant decrease in NGF protein levels, whereas there were no significant differences in NGF protein levels in control groups (Fig. 4). Although the precise mechanism of how vitamin D contributes to NGF release is unknown, these results demonstrate that short-term inhibition of the vitamin D-VDR pathway leads to a decrease in NGF release.

## Discussion

According to the Third National Health and Nutrition Examination Survey (USA), vitamin D deficiency frequently occurs in a wide range of populations, especially in the institutionalized and elderly [5], [26]. The misperception of vitamin D as a “simple vitamin” can hide the effects of its deficiency, which is a significant and on-going problem that has been termed the “silent epidemic”. Vitamin D deficiency has a number of potential consequences, many of which are still unknown [5], [27].

In a number of studies, it has been suggested that vitamin D in the brain regulates neurotrophic factor production, oxidative stress mechanisms, Ca^2+^ homeostasis and immune system functions [1], [2], [3], [4], [5], [6], [8], [10], [11], [12], [13], [14], [25]. To investigate the effects of short-term disruption of the vitamin D-VDR pathway on some of these mechanisms, the vitamin D receptor (VDR) gene was knocked down in cortical neurons using siRNAs. Following siRNA treatment, L-type voltage-sensitive calcium channels A1C (LVSCC-A1C), and -A1D (LVSCC-A1D) mRNA and protein levels and NGF production were determined in this study.

VDR was silenced by siRNAs because of the effects that potential compensatory mechanisms may have in VDR knock-out animals [28], which could interfere with detection of the direct effects of VDR silencing in neurons. Vitamin D deficiency can appear at any time throughout life, and compensatory mechanisms might not work well enough to adapt to vitamin D deficiency. Currently, there is no information about the effects that long-term vitamin D deficiency can have on the brain later in life due to the short life span of VDR knockout mice [29], [30]. It has been suggested that genetic ablation of VDR promotes premature aging in mice, and that vitamin D homeostasis regulates physiological aging [29]. Moreover, animals that have a transient-early vitamin D deficiency have relatively large lateral ventricles, reduced NGF protein and reduced expression of a number genes involved in neuronal structure. These observations demonstrate that transient hypovitaminosis D_3_ early in life not only disrupts brain development, but leads to persistent changes in the adult brain [14].

One of the most crucial changes that occurs in the aging brain is the disruption of calcium homeostasis [31]. The L-type voltage-sensitive Ca^2+^ channel, examined in *VDR* repressed neurons in the present study, is one of the most important proteins in calcium metabolism, aging and neurodegeneration. Previous studies have implicated Ca^2+^ dysregulation in the aging brain and Alzheimer's disease (AD), giving rise to the “Ca^2+^ hypothesis of brain aging and dementia” [32]. Increased LVSCC activity leads to up-regulation of Ca^2+^ related biomarkers in the aging hippocampus, and it has been implicated as one of the mechanisms related to this hypothesis. LVSCC down-regulation after vitamin D treatment in hippocampal and cortical neurons has previously been reported [2], [3], [25]. However, there is currently no information about the regulation of this channel under conditions of VDR repression. This study reports the effects of VDR silencing, on LVSCC-A1C expression, which has a substantial role in both aging and neurodegeneretion in primary cortical neuron cultures. Our results show that LVSCC-A1C mRNA levels were up-regulated in VDR siRNA-treated groups after both 12 and 24 hours of treatment, compared to the control groups. The same trend was observed when protein levels were analyzed. This marks the first time that up-regulation of LVSCC-A1C has been shown to be caused by VDR silencing. In addition, up-regulation of VDR and down-regulation of LVSCC-A1C and -A1D levels were observed in the vitamin D-treated VDR siRNA group. In other words, expression levels of both VDR and LVSCC-A1C in VDR siRNA-treated groups were normalized to the vitamin D-supplemented groups. These results support the notion that vitamin D regulates VDR expression in cortical neurons [33] and that vitamin D regulates LVSCC-A1C and -A1D expression in neurons [2], [3]. Our results also demonstrate that similar mechanisms operate in cortical and hippocampal neurons.

Regarding our results we may speculate that VDR, not the vitamin D membrane receptor, regulates LVSCC-A1C expression. It is also clear that LVSCC-A1C is a VDR-regulated protein. One of the most important results from this study was that VDR suppression did not affect LVSCC-A1D mRNA or protein expression. Although expression of this protein was altered by supplementation with vitamin D, this regulation might not be from VDR. As there are some clues that vitamin D triggers some signaling pathways by its membrane receptor (membrane-associated, rapid-response, steroid-binding protein; 1,25 MARRS) [15], we speculate that vitamin D may regulate LVSCC–A1D by that receptor. Thus, VDR-independent pathways, such as those dependent on 1,25 MARRS, should be further studied to clarify the action of this hormone.

Characterization of the short-term response of VDR-silenced neurons will provide novel data for guiding *in vivo* experiments to characterize the long-term effects of vitamin D-VDR pathway disruption. Based on our findings, we speculate that VDR knockout mice may not display all the effects that vitamin D has on vitamin D-regulated proteins because we determined that LVSCC-A1D may not be regulated by VDR even though it is regulated by vitamin D. Therefore, VDR and vitamin D membrane receptor 1,25 MARRS double knock-out mice may be used to clarify vitamin D action.

Together with its role in calcium metabolism, vitamin D has been suggested to have protective effects in the nervous system through regulation of NGF, glial derived neurotrophic factor (GDNF) and neurotrophins. Regulation of NGF expression by vitamin D has been demonstrated in several cell types [1], [6], [8], [11], [13], [14], [17], [34]. However, no information has been published regarding NGF regulation when VDR has been repressed. In the present study, there was a significant reduction in NGF levels after VDR siRNA treatment. NGF is regulated by a variety of molecules in addition to vitamin D [35]. In our study, NGF down-regulation was observed when VDR was silenced; this may provide a molecular explanation for previous *in vivo* results demonstrating that animals exposed to transient-early vitamin D deficiency had reduced NGF protein levels [14]. Interestingly, we have previously shown that after 48 hours of Aβ treatment, there was a decrease in VDR protein, and after 72 hours of treatment, there was a decrease in NGF secretion [25]. Together, with the results of this present study, we speculate that the reduction in NGF release in Aβ-treated cortical neurons may be caused by the depletion of VDR. Currently, it is not clear whether vitamin D-mediated regulation of NGF is caused by direct transcriptional regulation or by another mechanism related to NGF production or release. Our results demonstrate, for the first time, that silencing of the vitamin D receptor leads to down-regulation of NGF in “cortical neurons”, suggesting that inefficient utilization of vitamin D may lead to a reduction of NGF in neurons during the aging process.

The rapid increase in LVSCC-A1C expression and NGF down-regulation as a response to VDR silencing indicates that neurons could be vulnerable to aging and neurodegeneration when there is a long-term, or permanent, inefficient utilization of vitamin D. Because both Aβ [25] and VDR siRNA-dependent supression of VDR (current study) have very similar effects on neurons, we speculate that insufficient vitamin D levels in AD patients, in addition to depletion of VDR protein by Aβ, could give rise to a series of deleterious effects. When considering the role that vitamin D plays in calcium metabolism and neurotrophic factor regulation, vitamin D may be indispensable in the brain function. Knowledge regarding the status of the vitamin D membrane receptor protein in Aβ-treated neurons and in models that are both neurodegenerative and vitamin D deficient will help us better understand the action of vitamin D.

Previously, we showed that vitamin D has the potential to rearrange neuronal calcium homeostasis that has been broken by Aβ or inhibit the toxicity induced by Aβ [25]. Thus, vitamin D can protect neurons against neurotoxicity. In this study, LDH levels and the apoptotic index of VDR siRNA-treated group did not differ from control groups. This can be explained by the short time period of the treatments. This suggests that vitamin D has protective effects after 24 hours of treatment in primary cortical neurons.

Consequently, our study demonstrates that vitamin D-VDR pathway has important roles in brain. NGF, a neuronal survival molecule that can potentially be used to treat neurodegenerative diseases [36] is a VDR-regulated protein that is affected by VDR suppression. Our data support the “Ca^2+^ hypothesis of brain aging and dementia,” and suggest that vitamin D is a crucial part of this hypothesis. Furthermore, we speculate that some of the toxic effects induced by Aβ, including disruption of calcium homeostasis and dysregulation of NGF synthesis, may be caused by vitamin D deficiency and/or the inefficient utilization of vitamin D due to VDR protein depletion.

## Materials and Methods

### Preparation of primary cortical neuron cultures

The study was approved by the Animal Welfare and Ethics Committee of Istanbul University, number 23797/20.09.2006. The procedures involving experimentation on animal subjects were performed in accord with both the guidelines of Istanbul University, and with the National Research Council's guidelines for the care and use of laboratory animals.

**Figure 5:**
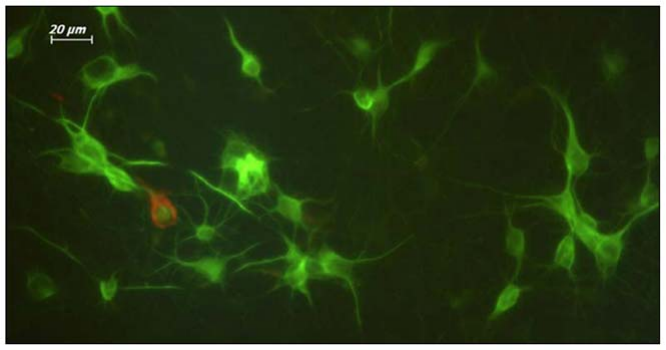
Seven-day-old primary cortical neurons were used to determine the neuron/glia ratio. The glia ratio was 10% in cultured cells. Neurons, green (FITC tagged PAN neuronal marker antibody); Glia, red (Texas Red (TR) tagged GFAP antibody). 40X magnification.

Neuronal cultures were prepared from the cerebral cortex of embryonic day 16 (E16) Sprague-Dawley rat embryos as previously described [25], [37]. Briefly, embryos were removed, and the cortex, excluding the olfactory cortex and the hippocampus, was dissected and freed of meninges. The cells were plated at a density of 6×10^5^ cells per dish in Leibovitz 15 (L15) media (GibcoBRL 11415-064, Invitrogen Inc., New York, USA) containing: 0.1 mg/ml conalbumin (Sigma C-7786, Sigma-Alderich Chemie GmbH, Steinheim, GE), 0.63 mg/ml sodium bicarbonate (GibcoBRL 25080-094, Invitrogen Inc., New York, USA), 0.1 mM putrescine (Sigma P-7505, Sigma-Alderich Chemie GmbH, Steinheim, GE), 10 ng/ml insulin (GibcoBRL 12585, Invitrogen Inc., New York, USA), 30 nM sodium selenite (Sigma S-5261, Sigma-Alderich Chemie GmbH, Steinheim, GE), 20 nM progesterone (Sigma P-6149, Sigma-Alderich Chemie GmbH, Steinheim, GE), 20 mM glucose (Sigma G-7021, Sigma-Alderich Chemie GmbH, Steinheim, GE) and 10 IU/ml PenStrep (Sigma P-4333, Sigma-Alderich Chemie GmbH, Steinheim, GE). These cells were then marked as L15+ and incubated for one day at 37°C and 5% CO_2_ in a humidified atmosphere. The next day, L15+ was replaced with neurobasal media, NBM (GibcoBRL 21103-049, Invitrogen Inc., New York, USA), containing 1:50 B-27 (GibcoBRL 17504-044, Invitrogen Inc., New York, USA), 10 IU/ml PenStrep, and 9% NaCl_2_ (Sigma S-3014, Sigma-Alderich Chemie GmbH, Steinheim, GE), and marked as NBM+. Cells were incubated for 7 days until the neurons extended neurites and became mature. The neuron/glia culture ratio was determined by immunofluorescent labeling with neuronal (Milli-Mark™ Pan Neuronal Marker, Millipore MAB2300., Millipore Corp., California, USA) and glial (GFAP, Invitrogen AB5804, Invitrogen Inc., New York, USA) markers, using Leica Application Suite Image Overlay Software (Leica Microsystems Ltd., Heerbrugg, GE) (Fig. 5).

### siRNA preparation and transfection

To determine the time it took to knockdown VDR mRNA and protein levels, three different time periods for VDR siRNA treatment (6, 12 and 24 hours) were tested in cortical neurons. Commercially available VDR siRNAs (ON-TARGET plus SMART pool Rat VDR, NM_017058 siRNA, Dharmacon L-097753-01-0010., Dharmacon Inc., Colorado, USA) were obtained from the manufacturer. Cyclophiline B siRNAs (ON-TARGET plus SMART pool Rat Cyclophiline B siRNA, Dharmacon D-001820, Dharmacon Inc., Colorado, USA) and non-target siRNAs (ON-TARGET plus SMART pool Rat Non-Target siRNA, Dharmacon D-001810, Dharmacon Inc., Colorado, USA) were used as the positive and negative controls, respectively. As additional controls, untreated cells and cells treated with only transfection reagent were used. DHARMAFECT® 3 (Dharmacon T-2003-02, Dharmacon Inc., Colorado, USA) transfection reagent was used for transfection. All transfections were performed on neurons that had been cultured for 7 days. After treatment, the transfection media was replaced with NBM+ including antibiotics. All RNA isolations were performed 24 hours after the applications.

### Vitamin D (1,25 (OH)_2_D_3_) application

To detect the effect of vitamin D on supressed VDR expression, the earliest time point after siRNA treatment was 12 hours. VDR siRNA-transfected cortical neurons (following 12 hours of VDR siRNA treatment) were incubated with vitamin D (1,25 (OH)_2_D_3_, Sigma C-9756, Sigma-Alderich Chemie GmbH, Steinheim, GE) for 12 hours. The final concentration of vitamin D (1×10^−7^ M) in culture was determined from the preliminary results.

### Cytotoxicity and cell death assay

Cytotoxicity levels were determined by measuring the amount of Lactate dehydrogenase (LDH) released into the culture media using a Cytotoxicity Detection Kit (Roche 11644793001, Roche Diagnostics GmbH Roche Applied Science Mannheim, GE) by ELISA according to the manufacturer's protocol. Each sample was tested in triplicate.

Cell death was determined using an In Situ Cell Death Detection Kit, Fluorescein (Roche 11684795910, Roche Diagnostics GmbH Roche Applied Science Mannheim, GE) according to the manufacturer's protocol. TUNEL-positive and TUNEL-negative cells were determined by overlaying fluorescent and phase contrast micrographs using Leica Application Suite Image Overlay Software.

### Quantitative-real time polymerase chain reaction (qRT-PCR)

RNA was isolated from cultured neurons using a High Pure RNA Isolation Kit (Roche 11828665001, Roche Diagnostics GmbH Roche Applied Science Mannheim, GE). cDNA was prepared using a Transcriptor First Strand cDNA Synthesis Kit (Roche 04379012001, Roche Diagnostics GmbH Roche Applied Science Mannheim, GE). VDR, LVSCC-A1C, LVSCC-A1D and Cyclophilin B mRNA levels in cortical neurons were analyzed using qRT-PCR, Universal Probe Library (UPL) probes, a Lightcycler 480 Probe Master Mix kit (Roche 04707494001, Roche Applied Biosystems™, California, USA) and a LIGHTCYCLER 480 (Roche Applied Biosystems™ California, USA). All primers and probes were chosen from Roche's UPL system as follows: VDR, NM_017058.1, UPL Probe #.65 (Roche 04688643001, Roche Applied Biosystems™, California, USA); LVSCC-A1C, NM_012517.2 UPL Probe #73 (Roche 04688961001, Roche Applied Biosystems™, California, USA); LVSCC-A1D, NM_017298.1 UPL Probe #82 (Roche 04689054001, Roche Applied Biosystems™, California, USA) and Cyclophilin B, NM_022536.1, UPL Probe #97 (Roche 04692144001, Roche Applied Biosystems™, California, USA). GAPDH (GAPDH Gene Assay, Roche 05046203001, Roche Applied Biosystems™, California, USA), Beta actin (ACTB Gene Assay, Roche 05046203001, Roche Applied Biosystems™, California, USA) and HPRT (HPRT NM_012583.2 UPL Probe #95, Roche 04692128001, Roche Applied Biosystems™, California, USA) were used as endogenous reference genes to normalize mRNA levels for qRT-PCR [38]. Five control sample serial dilutions were used to calculate the PCR efficiency. Reaction mixtures without cDNA template were utilized as negative controls. For each group, six dishes (6×10^5^ cells per dish) were used to extract mRNA. For each group, mRNA isolations were performed three times, and qRT-PCR was repeated two times for each sample in one experiment. The experiment was repeated three times.

### Western blot

Total protein extracts were prepared from 2×10^6^ cells with M-PER Mammalian Protein extraction reagent (Thermo Scientific 78501, Thermo Fisher Scientific Inc. Illinois, USA) according to the manufacturer's protocol. Prior to extraction, 1x Halt Protease Inhibitor Cocktail (Thermo Scientific 78429), 17 µg/ml Calpain Inhibitor I (Roche 11086090001, Roche Applied Biosystems™, California, USA) and 7 µg/ml Calpain Inhibitor II (Roche 11086103001, Roche Applied Biosystems™, California, USA) were added to the extraction reagent. Nuclear proteins were extracted from 2×10^6^ cells using NE-PER Nuclear and Cytoplasmic Extraction Reagent (Thermo Scientific 78833, Thermo Fisher Scientific Inc. Illinois, USA) according to the manufacturer's protocol. Prior to extraction, 1x Halt Protease Inhibitor Cocktail was added to the extraction reagent. Protein concentrations were assessed using a Picodrop™ (Picodrop Limited, Saffron Walden, UK). Briefly, 50 µg of proteins from cell homogenates were separated by electrophoresis through a precast Novex® 4–20% Tris-Glycine Gel (Invitrogen EC6028BOX, Invitrogen Inc., New York, USA). Proteins were then transferred to PVDF membrane using an IBlot Dry Blotting System (Invitrogen Inc., New York, USA). PVDF membranes were blocked with 5% dry milk over night at 4°C, incubated with primary antibodies over night at 4°C, then incubated in secondary antibodies for 2 hours at room temperature. PVDF membranes, containing proteins from nuclear extracts, were incubated with a vitamin D receptor rat monoclonal antibody (Chemicon, Millipore MAB1360, Millipore Corp., California, USA) at a 1∶2,000 dilution and further processed with a horseradish peroxidase (HRP)-conjugated goat anti-rat secondary antibody (Chemicon, Millipore AP136P, Millipore Corp. California, USA) at a 1∶3,000 dilution. All other PVDF membranes, including total protein extracts, were incubated with a LVSCC-A1C rabbit polyclonal antibody (Abcam Ab58552, Abcam Inc., Massachusetts, USA) and LVSCC-A1D rabbit polyclonal antibody (Abcam Ab54421, Abcam Inc., Massachusetts, USA) at a 1∶3,000 dilution, following incubation with HRP-conjugated goat anti-rabbit secondary antibody (Abcam Ab6721, Abcam Inc., Massachusetts, USA) at a 1∶3,000 dilution. A 1∶5,000 dilution of Beta actin rabbit polyclonal antibody (Abcam Ab8227, Abcam Inc., Massachusetts, USA) was used as a loading control. Signals were detected using a Lumi-Light^PLUS^ Western Blotting Substrate (Roche 12 015 196 001, Roche Applied Biosystems™, California, USA). Each sample was tested in duplicate, and western blots were repeated three times.

### NGF assay

The amount of NGF secreted into culture media was determined using a Chemikine Nerve Growth Factor (NGF) Sandwich ELISA kit (Chemicon CYT304, Millipore Corp., California, USA) by ELISA. Each sample was tested in triplicate, and each test was replicated.

### Statistics

Ct values obtained from qRT-PCR were calculated by the ΔCt = 2 ^(Geometric mean of reference genes - Ct target gene)^ formula for determining relative target gene expression levels. Microsoft Office Excel was utilized to perform the calculations and produce graphics. The results were compared with one-way ANOVA using GraphPad InStat DTCG 3.06 (GraphPad Software, Inc., San Diego, USA), and p<0.05 was considered to be statistically significant. Data are represented as mean standard deviations (SD).

A more sophisticated analysis for qRT-PCR results was performed using the Relative Expression Software Tool, REST 2008 (Corbett Research Pty LtD and Michael Pfaffl, New South Wales, AU), which has an algorithm for PCR efficiency, normalization and 95% confidence interval calculations. Reaction efficiencies were calculated using five different cDNA template dilutions. The software's hypothesis test, P(H1), determines the probability that the difference between two groups is due to chance. Thus, a P(H1) <0.05 indicates that there is a significant difference between the two groups, and the probability that this difference is due to chance is less than 5%. REST 2008 and ANOVA results were consistent with each other.

Comparisons of western blot band intensities were calculated using Image J 1.44a software (Wayne Rasband, National Institute of Health, USA). This program calculates the absolute intensity value for each protein band by measuring area and pixel values. The target protein absolute intensity relative to Beta actin absolute intensity ratio is defined as the “band intensities relative to Beta actin,” and was used to compare protein expression between each group. The relative protein expression of each group was compared using GraphPad InStat DTCG 3.06 one-way ANOVA method, and a p<0.05 was considered to be statistically significant. Data are presented as a mean SD.

Each ELISA sample was read three times, and the mean values were used in percentage calculations. Negative control mean values were subtracted from the sample and control group mean values. Cytotoxicity levels were compared to control groups. Calculations were made according to the formula given below, and Microsoft Office Excel was used to create the graphic: Cytotoxicity (%)  =  [(expected value – low control)/(high control - low control)] x100. NGF concentration were calculated using standard curve (R^2^ = 0.94) and the data were presented as %NGF. Raw data for each group was analyzed using the GraphPad InStat DTCG 3.06 one-way ANOVA method, and a p<0.05 was considered to be statistically significant. NGF data were given as the mean SD.
